# Redox-Activated Proton Transfer through a Redundant Network in the Q_o_ Site of Cytochrome *bc*_1_

**DOI:** 10.1021/acs.jcim.4c02361

**Published:** 2025-02-26

**Authors:** Guilherme M. Arantes

**Affiliations:** Department of Biochemistry, Instituto de Química, Universidade de São Paulo, Av. Prof. Lineu Prestes 748, 05508-900, São Paulo, SP, Brazil

## Abstract

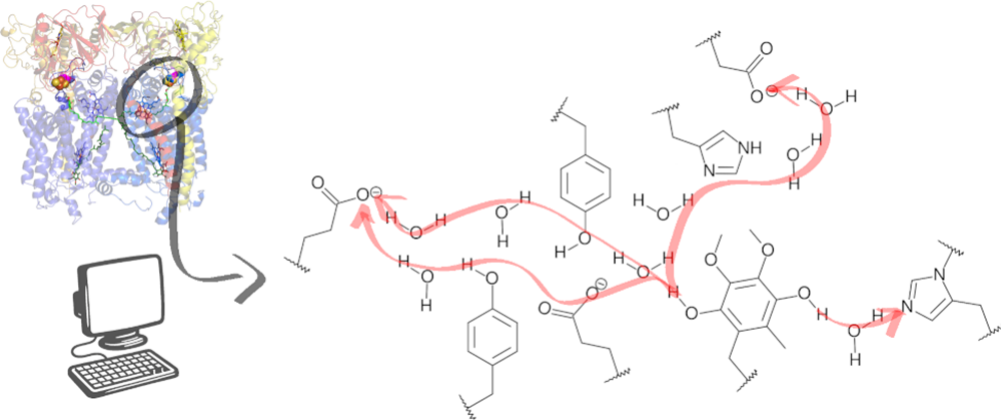

Proton translocation catalyzed by cytochrome *bc*_1_ (respiratory complex III) during coenzyme-Q redox cycling is a critical bioenergetic process, yet its detailed molecular mechanism remains incompletely understood. In this study, the energetics of the long-range proton transfers through multiple proton-conducting wires in the Q_o_ site of the *bc*_1_ complex was investigated computationally using hybrid QM/MM simulations and a specialized reaction coordinate. Key reactive groups and proton transfer mechanisms were characterized, confirming the propionate-A group of heme *b*_*L*_ as a plausible proton acceptor. Upon coenzyme-Q oxidation, a Grotthuss hopping mechanism is activated, facilitating proton transfer along three distinct pathways with comparable barriers and stability. These pathways operate redundantly, forming a robust proton-conducting network, and account for the unusual experimental behavior observed in single-point mutations. Energetic analyses exclude charged closed-shell species as likely intermediates and propose a reaction sequence for coenzyme-Q oxidation proceeding as QH_2_ → QH^•^ → Q^0^, either via coupled proton–electron transfers or stepwise mechanisms involving open-shell intermediates. These findings elucidate mechanistic details of the Q-cycle and improve our understanding of the catalytic reactions supporting redox-activated proton transfer in respiratory enzymes.

## Introduction

Cytochrome *bc*_1_, also known as respiratory complex III, is fundamental for cellular respiration and photosynthesis.^1^ It catalyzes the Q-cycle,^2−4^ where the membrane-soluble, two-electron carrier coenzyme-Q (here generically named Q) undergoes redox reactions that sustain the electron transport chain and translocate protons across the membrane. In addition to its primary role in energy transduction, the *bc*_1_ complex can generate reactive oxygen species, linking it to metabolic dysfunctions and stress.^5^ Therefore, the catalytic mechanism and the inhibition of the *bc*_1_ complex have important biomedical^6^ and biotechnological^7^ applications.

**Figure 1:**
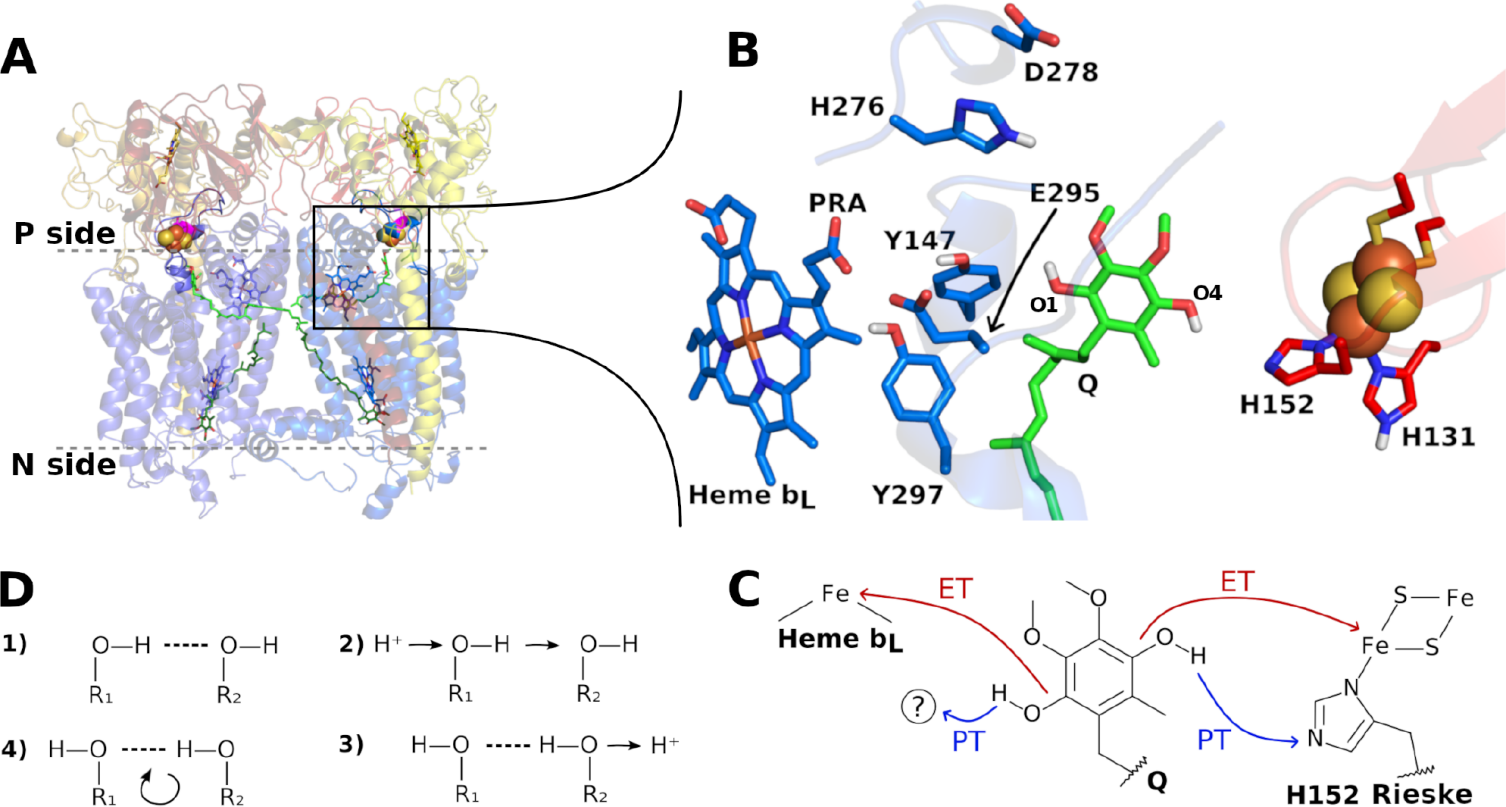
Structure of cytochrome *bc*_1_ and reactivity of its Q_o_ site. (A) Essential catalytic units in the *bc*_1_ dimer from *R. sphaeroides*(8) are shown in cartoon with cytochrome *b* (cyt *b*) in blue, cytochrome *c*_1_ in yellow, and Rieske protein in red. Membrane interface is in gray dashes with Q molecules in green sticks bound to the Q_o_ site (membrane P side) in quinol form and bound to the Q_i_ site (N side) in quinone form. FeS clusters are in orange and yellow spheres, hemes *b*_*L*_ (near site Q_o_) and *b*_*H*_ (near site Q_i_) in blue sticks, hemes *c* in yellow and cyt *b* D278 in magenta. (B) Close and rotated view of reactive groups in the Q_o_ site with labels in residues and in phenolic oxygens of the quinol substrate. (C) Electron (ET) and proton (PT) transfers for complete quinol oxidation. The question mark indicates that acceptors of one phenolic proton are unknown and investigated here. (D) Four steps of a Grotthuss proton hopping between two hydrogen-bonded groups.

The structure of the *bc*_1_ dimer is well-established (Figure 1A).^1,8,9^ The Q substrate binds to the Q_o_ active site in its reduced quinol form (dihydroquinone, QH_2_; Figure 1B). Extensive spectroscopic, kinetic, and mutational studies have characterized the adjacent heme *b*_*L*_ in the cytochrome *b* unit and [2Fe–2S] cluster in the Rieske protein as electron acceptors for quinol oxidation (Figure 1C).^9^ Electron transfer in the Q_o_ site is bifurcated, with each electron proceeding to a different metal center via quantum tunneling.^10−12^ Due to the stochastic nature of this process, it is unlikely that both transfers occur simultaneously, requiring an intermediate and transient semiquinol radical (one-electron oxidized).^9^

Two chemical protons are also released from quinol upon oxidation (Figure 1C). However, the groups within the Q_o_ site that participate in binding and transferring these protons to the bulk solvent on the positive side of the membrane remain poorly understood.^9,13^ Identifying these groups and elucidating the long-range proton transfer (PT) mechanism in the *bc*_1_ complex are the primary objectives of this work.

Long-range proton transfer in aqueous solutions and within solvated protein channels or cavities occurs through proton-conducting wires composed of water molecules and protonable groups connected by sequential hydrogen bonds (H-bonds, step 1 in Figure 1D).^14^ Originally proposed by Grotthuss,^15^ the conduction of an excess H^+^ through the wire involves a series of bond-breaking and forming events, or proton hops, along the wire (steps 2 and 3 in Figure 1D), followed by the reorientation of participating groups (step 4, returning to step 1 in Figure 1D). Thus, the composition of the proton wire and its interactions with the surrounding environment are critical for efficient proton transport.

The energetics of proton transfer along these wires can be estimated using molecular simulations with hybrid quantum chemical/molecular mechanical (QC/MM) potentials^16^ or related methods.^17^ The highly concerted Grotthuss hopping is effectively modeled using specialized reaction coordinates, such as the modified center of excess charge (mCEC),^18,19^ which capture the nonlinear, three-dimensional nature of proton wires and the nonlocal dynamics of the transfer process. Notably, this coordinate does not require prior identification of specific atom pairs involved in each transfer step. Instead, hopping sequences and the participation of particular groups naturally emerge from the simulated reaction. Solvation dynamics and interactions with the wire environment should be reasonably sampled, so an efficient quantum chemical (QC) treatment is also required.

In previous studies, we used extensive molecular dynamics (MD) simulations to characterize interactions within the Q_o_ site.^11,20^ These simulations identified high flexibility among side chains, leading to three binding modes for the quinol substrate. Two of these modes appear proximal to heme *b*_*L*_ and enable electron and proton transfer, while a third mode represents a prereactive state, in agreement with recent cryoEM structures with a bound Q.^21−23^ Additionally, our MD results show that the Q_o_ site is highly hydrated, with several water molecules creating an H-bond network connecting quinol with conserved residues such as Y147, E295, and Y297 (residue numbering from the *bc*_1_ complex of *R. sphaeroides*, Figure 1B). Through this network, at least five distinct proton wires were identified that could facilitate proton transport to final acceptors like H152, D278 and the heme *b*_*L*_ propionate-A group (PRA_*bL*_) that then release the protons to the bulk water.

Here, the energetics of proton transfer (PT) from the Q substrate in various redox states, across the multiple proton wires identified at the Q_o_ site, is evaluated to determine the relative stability and composition of the wires, as well as the sequence of proton hopping events. To simulate PT with greater accuracy, free energy profiles were obtained using hybrid QC/MM simulations and the mCEC reaction coordinate as mentioned above. The results confirm that heme PRA_*bL*_ can serve as a final proton acceptor via energetically favorable pathways, whereas D278 appears less likely to fulfill this role. Y147 is shown to be essential for complete PT, acting as an intermediary proton relay in all identified wires. The proton wires exhibit redundancy, functioning as a robust proton-conducting network resilient to structural fluctuations or single-point mutations. Based on these findings, a sequence of reactions constituting the Q-cycle at the Q_o_ site is proposed, which may also help to elucidate the Q redox chemistry in other respiratory enzymes.

## Methods

### Set-Up of Molecular Models

The cytochrome *bc*_1_ model used here to simulate PT reactions was based on the X-ray crystal structure of *Rhodobacter sphaeroides* (PDB 2QJP(24)) with ubiquinol-6 (with 6 isoprenoid units) bound to the Q_o_ site, and embedded in a solvated POPC lipid membrane. This model system was relaxed and equilibrated in previous classical MD simulations, where details of model construction were provided.^20^ Three configurations, representative of the local conformation and hydration of key residues in the Q_o_ site for the two reactive binding modes identified,^20^ were extracted from these trajectories at 147.0 ns from cyt *b* chain A of the dimer (referred to as 147A), at 141.0 ns (141D) and 152.9 ns (152D) from cyt *b* chain D (Table S1, Supporting Information) and treated equivalently herein for the PT simulations.

Each model was built by centering the initial geometry at the phenolic oxygen O1 of QH_2_ in the studied Q_o_ site. All atoms beyond *r*_cut_ = 32 Å, plus two iterations of bonded neighbors, were truncated, leaving approximately 15,800 atoms in the model. O1 in QH_2_ is positioned midway between the Rieske protein H152 and the cyt *b* heme PRA_*bL*_. In all simulations, atoms located further than *r*_move_ = 16 Å from O1, plus two iterations of bonded neighbors, were kept frozen, while the remaining atoms were relaxed for 5 ps of molecular dynamics with the QC/MM potential.^25−27^

**Figure 2:**
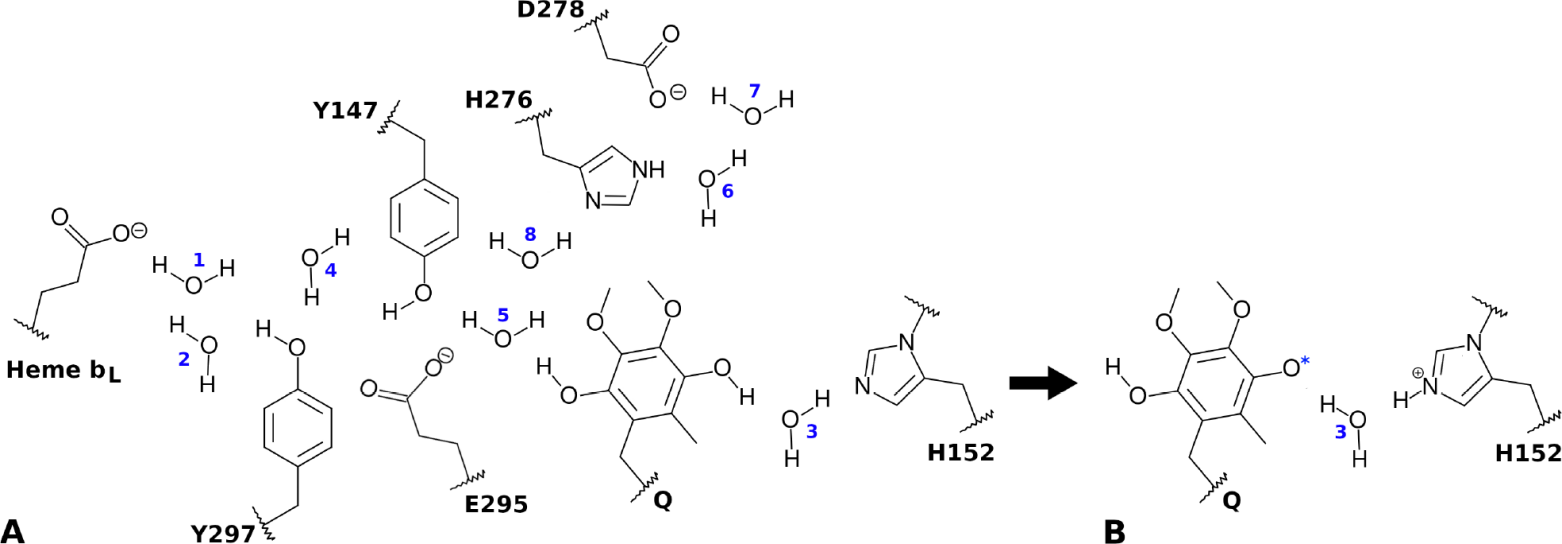
H-bond network and reactive centers in the Q_o_ site. For each QC/MM simulation, only groups and labeled waters given in Table 2 were represented in the QC region. (A) Reactant state simulated in Figure 3, corresponding to the state before the first PT. (B) Reactant state for the second PT simulated in Figures 4–6, after the first PT to H152 and with all other groups as shown in (A). The asterisk in O4 indicates that various oxidation states were simulated.

Simulated reactant states consisted of a combination of two protonation and three redox forms: Before the first PT, the Q substrate was modeled in the double-protonated form (QH2, Figure 2A), with the Rieske H152 N_ϵ_ deprotonated. After the first PT to H152 (Figure 2B), Q was in the monoprotonated form (QH) and H152 N_ϵ_ was protonated. The three Q substrate redox states were as follows: fully- or double-reduced Q with oxidized FeS center and heme *b*_*L*_ (Q_red_ state); one-electron oxidized Q radical with reduced FeS center and oxidized heme *b*_*L*_ (Q_semi_); and two-electron oxidized Q with reduced FeS center and heme *b*_*L*_ (Q_oxi_).

### Hybrid QC/MM Potential

Various QC regions were studied (Figure 2 and Table 2). The hydrophilic Q-head of Q was always modeled in the QC region and the hydrophobic Q-tail in the MM region, with the QC/MM boundary at the C7–C8 bond (first isoprenoid group). The side chain of Rieske H152 was also always in the QC region and its N_δ_–Fe bond was capped with a hydrogen link-atom, with the FeS center (including both iron and sulfide atoms) and its remaining ligands modeled in the MM region. For heme *b*_*L*_, only the PRA_*bL*_ group was in the QC region, capped at the C_α_–C2 bond. All protein side chains in the QC region had their boundary placed at the C_α_–C_β_ bond. Water molecules shown in Figure 2 were included in the QC region accordingly to their labels and Table 2. Extra water molecules were represented in the QC region in comparison to the minimum number of waters previously identified for each proton wire (Table 1).^20^

**Table 1:** Proton Wires Previously Identified^20^ for Transfer between the Quinol Donor and Various Acceptors in the Q_o_ Site, with Intermediate Residues and Water Molecules (Minimum Number: Labels Given in Figure 2) Required for Proton Hopping

wire	donor	residues	water	acceptor
A	Q_O4_		1:3	H152_Nϵ_
B	Q_O1_	Y147, E295,	1:1	PRA_*bL*,Oγ_
C	Q_O1_	Y147, Y297	2:1,5	PRA_*bL*,Oγ_
D	Q_O1_	Y147	3:1,4,5	PRA_*bL*,Oγ_
E	Q_O1_	Y147, E295, H276	3:5,6,7	D278_Oδ_

The efficient PM6 semiempirical method^28^ was used for the QC region. Comparison with higher levels of theory (DFT) suggests that this treatment is reasonably accurate (Figure S1, Supporting Information). For the MM region and Lennard-Jones parameters of QC centers in QC/MM simulations, the same force-field was applied as in previous classical MD simulations,^20,29,30^ using CHARMM36m^31^ and TIP3P^32^ parameters for protein, lipids and water, with recalibrated parameters for FeS ligands and heme *b* centers.^20^ Hydrogen link-atoms were used to cap valencies in the QC region.

For simulations with Q in the partially oxidized semiquinol form (Q_semi_), it was assumed that electron transfer proceeded to the FeS center, reducing the partial charge of FE2 (Cys-bound) by −0.50 |*e*| and each inorganic sulfide by −0.25 |*e*|. For simulations with Q fully oxidized (Q_oxi_), it was assumed both the heme *b*_*L*_ and the FeS center were reduced, reducing the partial charge of heme FE by −0.60 |*e*| and each bound nitrogen in the porphyrin ring by −0.10 |*e*|. These changes were determined after comparing partial charges in oxidized and reduced states from DFT calculations on model compounds for FeS and heme centers and were assigned before any QC/MM boundary charge redistribution.^33^ Given that these centers are spatially distant and separated by multiple covalent bonds from the reactive protons, any inaccuracies in their MM parameters are expected to have a minimal impact on the simulation results.

Standard electrostatic QC/MM embedding was used, with long-range electrostatics cut off by an atom-based force-switching function (*r*_on_ = 8 Å and *r*_off_ = 12 Å). This cutoff aligns with the model setup, ensuring that all moving atoms remain within the switching range of long-range interactions, *r*_off_ < (*r*_cut_ – *r*_move_). To prevent diffusion into the MM region, a flat-bottom harmonic potential 2.0 Å wide with a 150 kJ mol^–1^ Å^–2^ force constant was applied to the oxygens of all QC waters, centered at their initial positions. All QC/MM simulations and analysis were conducted with the pDynamo library version 3,^33,34^ where the mCEC^18^ reaction coordinate was implemented (see below). Initial configurations and sample pDynamo scripts with all simulation parameters are available online^35^ to enable full reproduction of this study.

### Umbrella Sampling and Reaction Coordinate

Proton transfer reactions were simulated with these models and QC/MM potential by umbrella sampling (US)^36^ with Langevin MD, temperature of 310 K, collision frequency of 25.0 ps^–1^ and a time-step of 1 fs. Umbrella sampling was performed along the ζ reaction coordinate defined below (eq 2). The ζ = [0,1] interval was divided in equally spaced windows of 0.1 length and a force constant *k*_umb_ = 1000 kJ mol^–1^ Å^–2^ was employed. If necessary, additional windows were included to increase ζ overlap with *k*_umb_′ = 3*k*_umb_. Each window was sampled by at least 0.5 ns, which is shown in Figure S2 to be enough for convergence of the profiles. Some simulations had sampling increased to 1 ns per window as indicated. Free energy profiles were pieced together using the weighted histogram analysis method (WHAM)^37^ with the initial 0.1 ns of each US window discarded for equilibration. Statistical uncertainties were estimated as 95% confidence intervals by bootstrap analysis with 30 resampling cycles.^38^

The modified center of excess charge (mCEC)^18,19^ was implemented in the pDynamo 3 library and used here as a reaction coordinate to drive global and nonlinear proton-transfer in Grotthuss proton hopping (Figure 1D) through the identified wires. Briefly, the mCEC is defined as
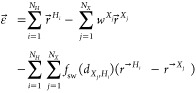
1where *X*_*j*_ represents the atom bound to a ionizable proton (*H*_*i*_), *r⃗*^*X*_*i*_^ is the position of *X*_*i*_ and  is the weight associated with atom *X*_*j*_. This is defined by the least number of protons bound to that atom. *f*_sw_ is a smooth switching function depending on A to B atom distance (*d*_A,B_). Here, values of *r*_sw_ = 1.20 Å and *d*_sw_ = 0.04 Å were used in the *f*_sw_ function.^18^ Weights were assigned as *w*_Tyr_^O^ = 1.0, *w*_Heme,Asp,Glu_^O^ = 0.0, *w*_His_^N^ = 0.5, *w*_water_^O^ = 2.0 and *w*_Q_^O^ = 0.0.

**Figure 3:**
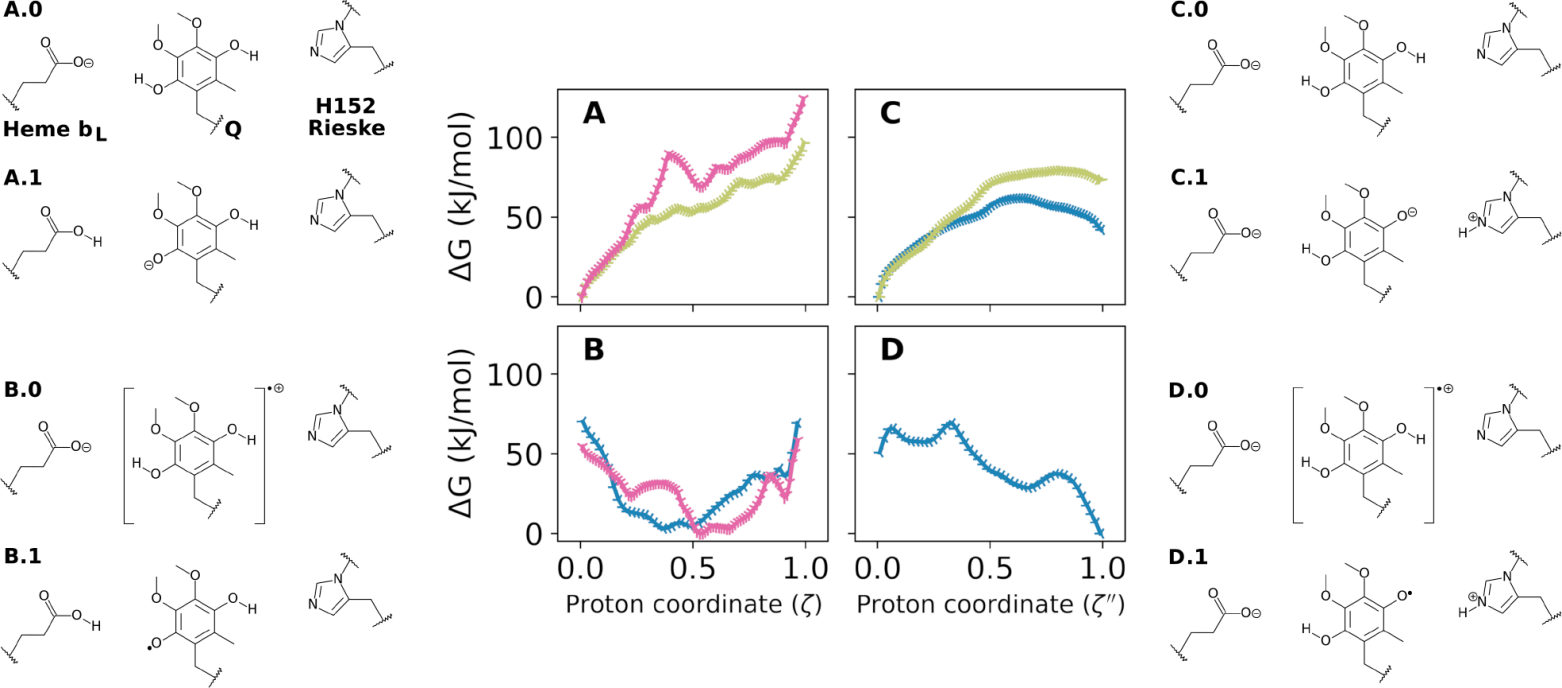
Reactivity of the first proton transfer from the quinol substrate depicted as free energy profiles. PT was simulated from double-protonated and: (A) double-reduced quinol (QH_2_) to PRA_*bL*_; (B) semiquinol radical () to PRA_*bL*_; (C) double-reduced quinol (QH_2_) to H152; and (D) semiquinol radical () to H152. Charge states of PRA_*bL*_, Q and H152 groups in the reactant (proton coordinate ζ = 0 → *X*.0) and product (ζ = 1 → *X*.1) are shown besides the respective profile *X*. Simulations were performed with hybrid QC/MM potentials with QC region defined in Table 2 and Figure 2A. Profile colors denote different wires and initial configurations with 147A in blue, 141D in gold and 152D in pink. Statistical uncertainties in the free energies are approximately ±1 kJ/mol and are smaller than the symbols shown in each panel.

**Figure 4:**
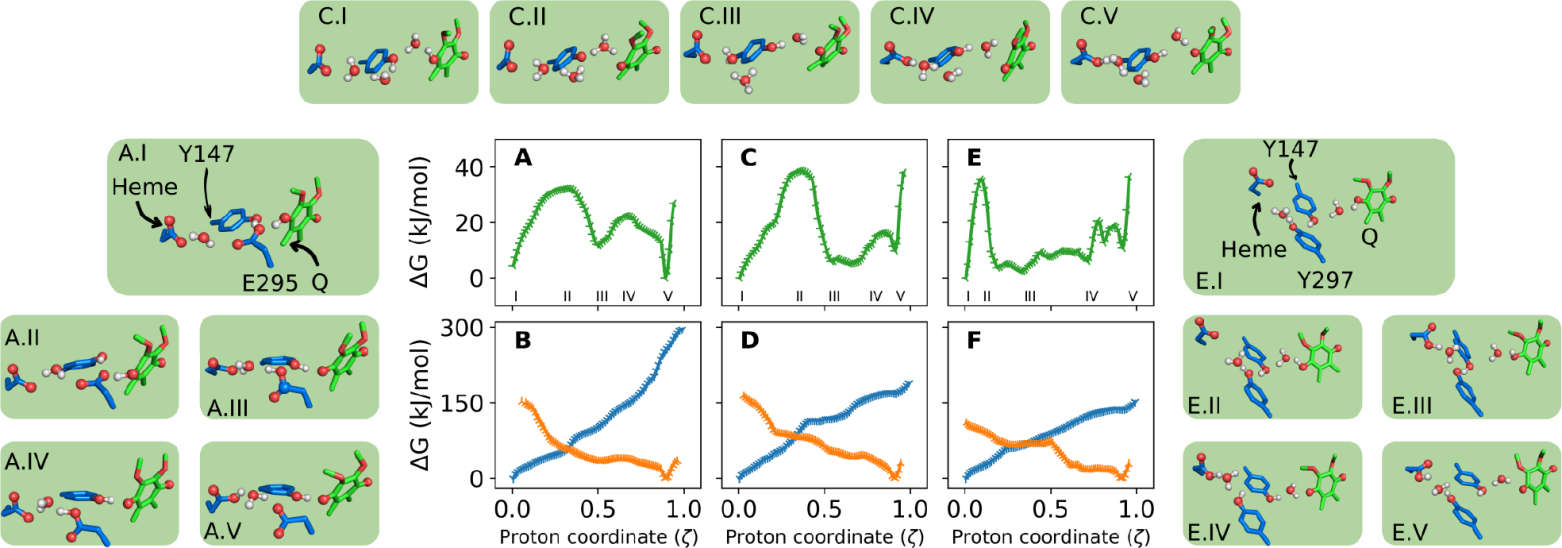
Reactivity of the second proton transfer to heme A-propionate (PRA_*bL*_) depicted as free energy profiles. Three different proton-wires from monoprotonated QH (Figure 2B) were simulated via: (A, B) Y147 and E295 (wire B in Table 1); (C, D) Y147 only (wire D); and (E, F) Y147 and Y297 (wire C). Colors denote the redox state of QH, with semiquinol (QH^•^) in green, reduced (QH^–^) in blue and fully oxidized (QH^+^) in orange. Boxed inserts show representative structures for reactive groups at proton coordinates indicated by roman numerals in the respective plot *X*-axis. For example, inset A.III corresponds to the intermediate found at ζ = 0.5 in panel (A).

**Figure 5:**
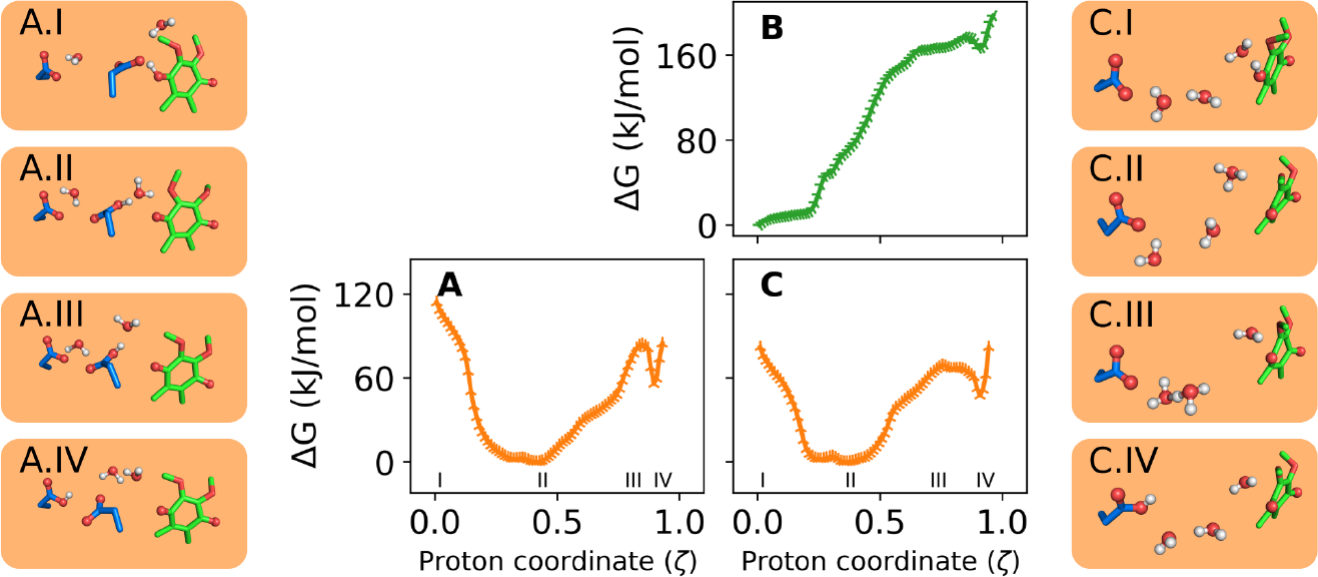
Reactivity of the second proton transfer to PRA_*bL*_ without an intervening Y147. Two proton-wires were simulated: (A) PT via E295 (wire B without Y147); and (B, C) PT via water only (wire D without Y147). Colors denote the QH redox state with semiquinol (QH^•^) in green and fully oxidized (QH^+^) in orange.

**Figure 6:**
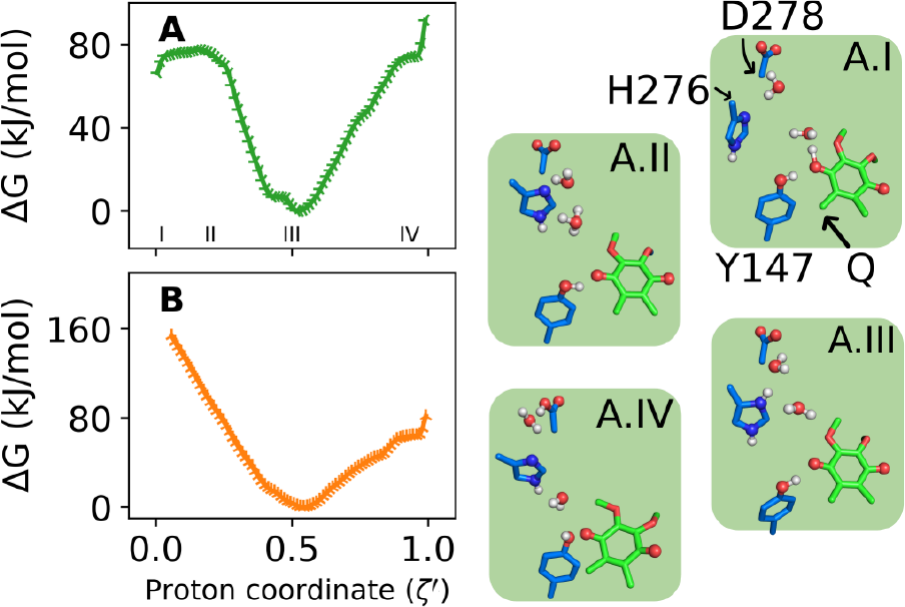
Reactivity of the second proton transfer to D278. One proton wire (E, Table 1) was simulated. Colors denote the QH redox state with semiquinol (QH^•^) in green and fully oxidized (QH^+^) in orange.

As the center of charge ε⃗ corresponds to a spatial (vector) coordinate, we employ the relative coordinate ζ to enhance sampling in US simulations:
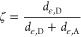
2where *d*_ε,D_ and *d*_ε,A_ are distances between ε⃗ and the position of the proton donor or the acceptor atoms, respectively. ζ is defined in the interval [0,1], with 0 corresponding to the reactant state (preproton transfer) and 1 is the product state (postproton transfer). In QC/MM simulations performed here, this type of reaction coordinate described transfer for different donor (D) and acceptor (A) pairs: from donor Q_O1_ to acceptor heme PRA_*bL*,Oγ_ (ζ coordinate) in Figures 3A,B, 4, and 5; from Q_O1_ to D278_*O*δ_ (ζ′ coordinate) in Figure 6; and from Q_O4_ to H152_Nϵ_ (ζ″ coordinate) in Figure 3C,D. All QC waters and protonable groups listed in Table 2 were included in the respective definition of mCEC coordinate.

**Table 2:** Details of the QC Region in Hybrid QC/MM Simulations^a^

figure	initial	QC groups	QC waters	QC(charge, multi)
3A	141D	Q, H152, PRA_*bL*_, Y147, E295	3:1,3,5	(−2, 1)
3A	152D	Q, H152, PRA_*bL*_, Y147	5:1,3,2,4,5	(−1, 1)
3B	152D	Q, H152, PRA_*bL*_, Y147	5:1,3,2,4,5	(0, 2)
3B	147A	Q, H152, PRA_*bL*_, Y147, Y297	3:1,3,5	(0, 2)
3C	147A	Q, H152, PRA_*bL*_, Y147, Y297	3:1,3,5	(0, 1)
3C	141D	Q, H152	1:3	(0, 1)
3D	147A	Q, H152, PRA_*bL*_, Y147, Y297	3:1,3,5	(0, 2)
4A	141D	Q, H152, PRA_*bL*_, Y147, E295	3:1,3,5	(−1, 2)
4B	141D	Q, H152, PRA_*bL*_, Y147, E295	3:1,3,5	(−2, 1), (0, 1)
4C	152D	Q, H152, PRA_*bL*_, Y147	5:1,3,2,4,5	(0, 2)
4D	152D	Q, H152, PRA_*bL*_, Y147	5:1,3,2,4,5	(−1, 1), (+1, 1)
4E	147A	Q, H152, PRA_*bL*_, Y147, Y297	3:1,3,5	(0, 2)
4F	147A	Q, H152, PRA_*bL*_, Y147, Y297	3:1,3,5	(−1, 1), (+1, 1)
5A	141D	Q, H152, PRA_*bL*_, E295	3:1,3,5	(0, 1)
5B	152D	Q, H152, PRA_*bL*_	5:1,3,2,4,5	(0, 2)
5C	152D	Q, H152, PRA_*bL*_	5:1,3,2,4,5	(+1, 1)
6A	141D	Q, H152, Y147, E295, H276, D278	5:3,5,6,7,8	(−1, 2)
6B	141D	Q, H152, Y147, E295, H276, D278	5:3,5,6,7,8	(0, 1)

a The figure column shows the corresponding figure and panel in the Results. Initial is the configuration used to start simulations (Table S1). QC groups and QC water molecules (total number:labels given in Figure 2) represented in the QC region. QC(charge, multi) gives the total charge and spin multiplicity in the QC region.

## Results

A set of 21 free energy profiles of proton transfer reactions in the Q_o_ site is presented here probing various proton wires (Table 1)^20^ and reactant states (Tables 2 and S1) for quinol oxidation. First, the acceptor group for the initial PT is identified, along with its product which serves as a reactant state for the subsequent second PT. Then, proton wires and acceptors for the second PT are tested to define the final secondary acceptor, the reactive composition of wires, and the expected proton hopping sequence. Detailed reaction mechanisms, describing bond breaking and formation sequences, will be presented for a select subset of reactions, particularly those with favorable energetics (e.g., Figure 4A) and thus more likely to occur.

### First Proton Transfer Proceeds to His152 and Generates a Monoprotonated Semiquinol Radical

The initial PT from the fully protonated quinol substrate was simulated and the estimated free energy profiles are shown in Figure 3. Using fully reduced QH_2_ as the donor and heme PRA_*bL*_ as the acceptor, Figure 3A shows steep uphill profiles with high free energies (over 100 kJ/mol) and no barrier for reverse PT, revealing an unstable product. This indicates that PT is unlikely for this reactant state and proton wires. Profiles are qualitatively similar for the two simulations, supporting that results are consistent across wires with different composition and initial configuration.

Figure 3B shows profiles for the same donor–acceptor pair, but now starting from a semiquinol radical reactant (), with one-electron oxidized by the high-potential Rieske FeS center. A pronounced free energy minimum (∼50 kJ/mol) is observed at proton coordinate ζ ∼ 0.5, corresponding to the transferred charge being trapped as a hydronium (H_3_O^+^) H-bonded to Y147 and the PT reaction does not complete to heme PRA_*bL*_. Again, profiles are qualitatively similar for PT through two different wires and initial configurations.

Figure 3C,D are the only plots in this study describing PT to Rieske H152, from donor Q_O4_ to acceptor H152_Nϵ_ connected by a water molecule in bridge, simultaneously H-bonded to both centers (Figure 2A and proton wire A in Table 1). The profiles in Figure 3C for PT from QH_2_, thus before any ET from the substrate, show a high barrier (>60 kJ/mol) and an unstable product, suggesting again that PT should not be observed before quinol oxidation.

Figure 3D was obtained for PT from  to H152, after one-electron oxidation of the quinol substrate by the FeS center. The profile shows a small barrier (<20 kJ/mol) and high stability (<−50 kJ/mol) for PT, suggesting that reaction would be fast and thermodynamically favorable. The simulated reaction mechanism for this PT is simply a proton donation from Q_O4_ to the bridge water oxygen, which in concert donates another proton to H152_Nϵ_, similar to a water asymmetric bond-stretch. The transition state at ζ*″* = 0.35 corresponds to a transient hydronium ion formed along this concerted transfer.

Thus, the only feasible and efficient reaction identified for the initial PT from quinol involves transfer from the one-electron oxidized  to protonate H152_Nϵ_. This reaction produces a monoprotonated semiquinol radical QH^•^.

### Second Proton Transfer Proceeds to the Propionate in Heme *b*_*L*_ by Three Different Wires

All simulations for the second PT in the Q_o_ site begin from a reactant state with the monoprotonated QH species (proton bound to O1, with O4 ionized), as shown in Figure 2B.

The free energy profiles in Figure 4 describe PT to the heme PRA_*bL*_ through three different proton wires (labeled B, C, and D in Table 1), involving E295, Y297, and Y147 side chains and nearby water molecules.^20^ All possible QH redox states are tested and the behavior for each state is qualitatively similar across the three wires.

If the reactant is fully reduced (QH^–^, blue profiles), the PT reaction free energies exceed 150 kJ/mol, indicating that the second PT would not occur, consistent with results from Figure 3A,C for the first PT. This outcome strongly indicates that proton transfer from Q is unlikely to proceed in the Q_o_ site without prior or concerted electron transfer.

In the double-oxidized reactant (QH^+^, orange profiles), the free energy for PT is barrier-less and downhill, leading to a stable, protonated heme *b*_*L*_ product (ζ = 0.9). However, the released “hot-proton” would transfer to any protonable group, making it highly improbable for the native Q-cycle to rely on such an irreversible and highly dissipative (exergonic) reaction.

Conversely, in the one-electron oxidized form (QH^•^, green profiles), low barriers (25–40 kJ/mol) emerge at early proton coordinates, leading to a protonated heme PRA_*bL*_ product, in a nearly equilibrium or thermoneutral reaction (Δ*G*_R_ = ±10 kJ/mol). This suggests a conservation of free energy and a reversible transformation,^39,40^ supporting a much more plausible pathway for the Q-cycle.

For the proton wire B via Y147 and E295 (Figure 4A and A.I–A.V insets), PT proceeds from QH^•^ with an early barrier (A.II) corresponding to breaking the O4–H bond and forming an intermediate protonated at the E295 side chain (A.III). The proton bound to E295 may come either directly from Q_O4_ or passed concertedly to Y147_OH_ which transfer the excess proton to E295. This intermediate is deprotonated by a bridging water passing the excess proton to heme (A.V).

In wire D, the role of E295 as an intermediate proton acceptor is substituted by water molecules (Figure 4C and C.I–C.V insets) that help to bridge PT from QH^•^ to Y147 (C.II), form a hydronium intermediate (C.III) instead of a protonated E295 and transfer the excess charge to heme (C.IV).

For the proton wire C via Y147 and Y297 (Figure 4E and E.I–E.V insets), the early barrier corresponds to formation of an extended H-bond network involving Q_O4_, the two Tyr_OH_ and the oxygens of two water molecules (E.II). An intermediate with one proton shared between Y147 and Y297 is formed with heme PRA_*bL*_ already protonated (E.III). Complete PT is achieved after rearrangement of the H-bond pattern (E.IV), reminiscent of a proton-hole transfer.^41^

### Residue Y147 Is Necessary to Complete the Second Proton Transfer

To investigate the chemical role of Y147 in the second PT reaction, simulations shown in Figure 5 were conducted using the same proton wires (B and D, Table 1), but with an unreactive Y147 now modeled in the MM region (Table 2). This mimics the effect of a Y147 point mutation to a residue unable to exchange protons, but with minimal disruption to the surrounding chemical environment. Y147 in the MM region can not form covalent bonds but can still form H-bonds and interact electrostatically with groups in the Q_o_ site.

The free energy profile in Figure 5A shows a striking difference compared to that in Figure 4B for the proton wire B. The initial PT from oxidized QH^+^ is again highly exergonic, forming a protonated E295 (inset A.II). However, the PT reaction does not complete to heme PRA_*bL*_ due to a substantial barrier (95 kJ/mol, A.III) and the instability of the protonated PRA_*bL*_ (A.IV). As a result, the excess proton becomes trapped in E295 when Y147 is chemically inactive. A similar pattern occurs in Figure 5C (wire D as in Figure 4D), but here trapping involves a hydronium ion (inset C.II). For reaction of the singly oxidized QH^•^ (Figure 5B), the free energies for PT to heme are over 160 kJ/mol, preventing any reaction. This outcome contrasts sharply with the PT behavior through the same wire when Y147 is chemically active (Figure 4C).

### Transfer to D278 Is Not Energetically Feasible

A recent study proposed that H276 and D278 in cyt *b* may function as the proton acceptors for the second PT.^42^ Hydration of the Q_o_ site revealed a network of H-bonds connecting the Q substrate to H276 and D278 in cyt *b* via 2 or 3 water molecules (Figure 1B and wire E in Table 1),^20^ supporting a Grotthuss proton hopping mechanism.

The energetics of this proton wire E is shown in Figure 6. For the semiquinol QH^•^ reactant, transfer proceeds from Q_O4_ by a small barrier (10 kJ/mol) via an Eigen-type ion (A.II), leading to a stable intermediate with the H276 side chain double-protonated (A.III). Proton transfer to D278 occurs through a water bridge but requires a high free energy (80 kJ/mol) and results in an unstable product (A.IV). For the double oxidized QH^+^ reactant, an analogous intermediate forms after a downhill transfer, with the excess proton again becoming trapped in H276.

## Discussion

A comprehensive set of reactant states, proton wires, and simulation set-ups for possible PT reactions in the Q_o_ site of cytochrome *bc*_1_ was investigated by hybrid QC/MM simulations and a specialized reaction coordinate (mCEC).

The statistical uncertainties for the calculated free energies are approximately ±1 kJ/mol. This represents a lower bound of the total uncertainty, which is challenging to assess due to the approximate nature of the simulations. The QC level employed in the QC/MM potential (see Methods) is one possible source of error. However, Figure S1 shows that this treatment is reasonably accurate compared to higher levels of theory (DFT), indicating a minor contribution to overall uncertainty.

Another possible concern is the use of configurations sampled in the QH_2_ reactant state (Table S1) as starting structures for the simulations of the second PT (Figures 4–6). However, this should not be a limitation, as the first ET and PT reactions are rapid (nanosecond time scale or faster) and no significant structural rearrangements are likely to occur within such a short period. Furthermore, the initial 100 ps of each US simulation window was discarded before estimating the free energy profiles, allowing sufficient time for relaxation and equilibration of the Q-head, side chains and water molecules in response to changes in reactant states.

While other sources of error may still be present (e.g., in the molecular model setup), the results presented here remain consistent across different models and initial configurations, providing strong qualitative reliability. Therefore, all discussions and conclusions will emphasize qualitative and relative trends, such as uphill vs downhill profiles, small vs large barriers, and complete vs trapped reactions.

**Figure 7:**
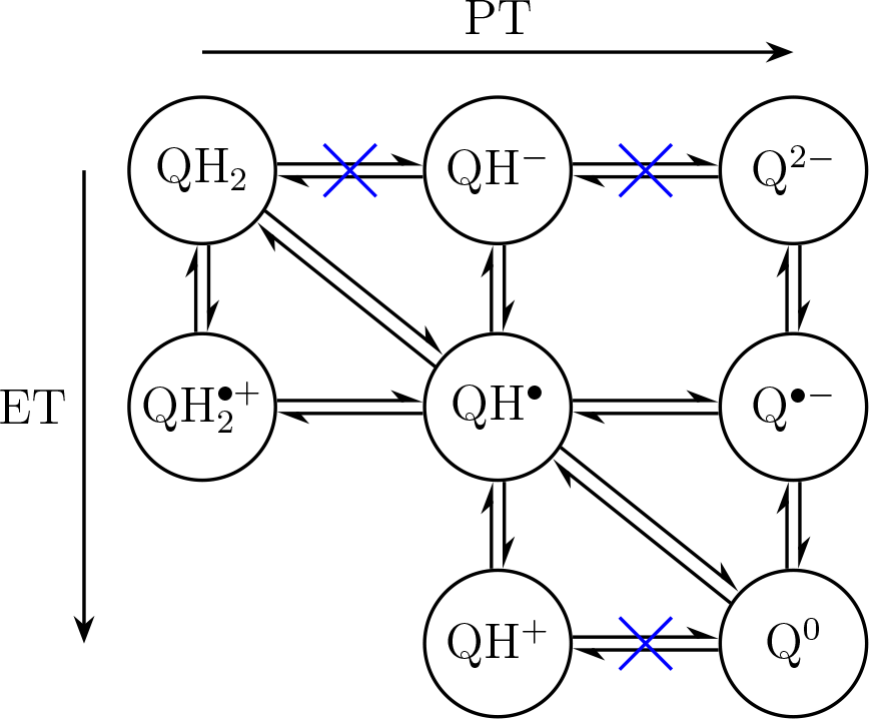
Possible reactions for quinol oxidation. Horizontal equilibria represent proton transfer (PT) reactions, which were investigated here. Vertical equilibria denote electron transfer (ET) and diagonal indicate coupled proton–electron transfer (CPET). Equilibria marked with a blue cross are unlikely to occur in the Q_o_ site under normal operation of the Q-cycle based on the analysis presented here.

Analysis of free energy profiles presented below (after Figure 7) is based on the following (bio)energetic rationale: highly uphill profiles (>70 kJ/mol) imply that the process is energetically unfeasible. Low barriers (<15 kJ/mol) for the reverse reaction indicate an unstable product. Trapped intermediates suggest that the transfer may remain incomplete, allowing the accumulation of intermediates, with possible reversibility or short-circuiting of the reaction. In these three cases, the simulated process or step is unlikely to occur or reach completion under physiological operation of the Q-cycle.

Moreover, steep downhill profiles suggest that a transfer would occur indiscriminately, with a “hot-proton” transferred to any acceptor group. These profiles dissipate substantial free energy, often exceeding what is provided by complete QH_2_ oxidation in the *bc*_1_ complex (∼70 kJ/mol)^9^ and again, the respective transfer step is unlikely to occur physiologically.

Plausible PT steps for the Q-cycle, however, should exhibit profiles with low to medium barriers (up to ∼45 kJ/mol) and operate near equilibrium or thermoneutral conditions (reaction |Δ*G*_R_| < 20 kJ/mol), with both conditions also required for the Q-cycle to operate in reverse over the same steps.

Figure 7 shows possible ET and PT reactions for complete quinol oxidation (or quinone Q^0^ reduction in reverse). Horizontal equilibria describe PT reactions, which were all tested here in the Q_o_ site for different proton acceptors (H152, PRA_*bL*_ and D278).

For the first PT (left to middle column in Figure 7), the reaction does not proceed to heme PRA_*bL*_ or to H152 before one-electron oxidation of quinol by the FeS center (Figure 3). After the first ET, PT still does not proceed to PRA_*bL*_ but transfer to H152 is fast and thermodynamically favorable.

Previous computational studies have shown that a coupled proton–electron transfer (CPET) from quinol to H152 should also be efficient.^11,12^ These studies included a FeS center bound to H152 within the QC region and also yielded a stable semiquinol radical (QH^•^) as the product.

Therefore, either a stepwise (in the ET + PT sequence, but not in PT + ET) or a coupled (CPET) transfer from QH_2_ to protonate H152 are possible and yield a monoprotonated semiquinol radical (QH^•^) for the first oxidation step (Figure 7). The second PT from Q_O1_ will proceed preferably once Q_O4_ is ionized and the substrate has undergone (one-electron) oxidation. These results are in line with previous experimental spectroscopic^9,43,44^ and mutational^45^ studies that suggested H152 is one of the proton acceptors.

Participation of heme PRA_*bL*_ as a proton acceptor was recently proposed,^20^ based on MD simulations of local hydration in the Q_o_ site and the formation of an H-bond network connecting QH_2_ and PRA_*bL*_, also supported by cryoEM structures.^21−23^ Involvement of propionate groups from redox-active hemes in PT has been suggested in other bioenergetic enzymes, including respiratory complex IV (CcO).^46−48^ However, obtaining experimental evidence for propionate participation in *bc*_1_ is challenging, as mutations affecting heme groups often render the enzyme nonfunctional, and may even prevent correct protein folding.^49^

One goal of this study was to determine whether the energetics of PT to PRA_*bL*_ is consistent with the proposed role of this group as an acceptor. Indeed, simulations of the second PT to heme PRA_*bL*_ through three different proton wires confirm this (Figure 4A,C,E, corresponding to QH^•^ ⇌ Q^•^^–^ in Figure 7). Barriers between 25 and 40 kJ/mol and reaction free energies of ±10 kJ/mol were obtained for all three wires, indicating that this is a thermodynamically feasible and kinetically fast PT pathway. This supports the viability of heme PRA_*bL*_ as an acceptor for the second proton in the Q_o_ site.

These barrier heights also suggest that the lifetime of the QH^•^ radical intermediate is on the order of tens of nanoseconds. Thus, experimental detection of this intermediate under equilibrium conditions, which has been debated extensively in the literature,^9^ would require ultrafast spectroscopic techniques capable of capturing species within this time scale.

Acidity of QH^•^ in the Q_o_ site is markedly higher than that of QH_2_, similar to what was found from solution measurements.^50^ Notably, the nearly thermoneutral reaction energy of the QH^•^ ⇌ Q^•^^–^ equilibria suggests that both species will be present in the Q_o_ site, with the lifetime of the deprotonated radical depending on its ET rate. The thermoneutral reaction further indicates that this PT step can proceed in reverse, consistent with observations from in vitro experiments on reconstituted^39^ and cofactor knockout^40^*bc*_1_ complexes.

A novel and interesting finding for the *bc*_1_ enzyme is that the three wires (B, C, and D) exhibit similar energetics and could potentially function with redundancy, forming a *proton conducting network*. This is also consistent with single-point mutations in residues E295 or Y297, that still yield functional *bc*_1_ complexes.^45^ Thus, the operation of multiple proton wires, along with the flexibility within the Q_o_ site leading to variations in side chain conformations and two reactive binding modes of the Q-head,^20^ could all contribute a significant entropic component that enhances PT efficiency in the Q_o_ site.

The three proton wires (B, C, and D) share two common features: the involvement of Y147 as an initial and transient proton acceptor and the delivery of the excess charge to heme PRA_*bL*_ via a water molecule. If Y147 is not chemically involved, neither PT from semiquinol QH^•^ or from fully oxidized QH^+^ results in complete transfer to heme PRA_*bL*_ (Figure 5). Thus, the role of Y147 as a transient proton relay (as observed in models A.II, C.II, and E.III of Figure 4) is critical for enabling an energetically favorable and complete PT to heme *b*_*L*_. Single-point mutations in Y147 have been shown to abolish or significantly impair oxidase activity in the Q_o_ site.^51^

A recent mutational study proposed^42^ that one of the chemical protons from quinol oxidation is transferred by H276 to D278 in cyt *b*. These residues are part of the previously identified H-bonded network within the Q_o_ site and the proton wire E (Table 1).^20^ However, PT from QH^•^ or QH^+^ does not proceed completely to D278; instead, the excess charge becomes trapped at H276 (Figure 6). H276 is also unlikely to act as the proton release group due to the high free energy barrier for its deprotonation to water and its relatively distant position from bulk water. Additionally, both H276 and D278 show low conservation among *bc*_1_ variants and are frequently mutated.^20^ Therefore, it can be concluded that H276 and D278 are unlike to function as acceptors for the second chemical proton released during quinol oxidation.

For the forward quinol oxidation in the Q_o_ site, the results presented here (Figures 3A,C and 4B,D,F), when analyzed using the above bioenergetic rationale, indicate that the equilibria marked by a blue cross in Figure 7 are unlikely to occur under physiological conditions. The reverse PT from H152 and QH^•^ to reform  is energetically unfavorable and kinetically slow. Therefore, if the same reaction steps obtained for the forward cycle are mirrored in the reverse Q-cycle operation, the coupled CPET (diagonal) reaction likely represents the most probable equilibrium between the left and middle columns in Figure 7.^11,12,44^

This analysis indicates that only neutral closed-shell Q species (first and third rows in Figure 7) are formed during both forward and reverse oxidation in the Q_o_ site. Double oxidation of a doubly protonated species (resulting in ) is highly improbable and was not considered here. Protonation reactions involving QH^+^, QH^–^, and Q^2–^ are highly uphill or excessively dissipate free energy, depending on the direction considered.

Therefore, the most plausible reaction sequence for quinol oxidation in the Q_o_ site is QH_2_ → QH^•^ → Q^0^, occurring either via coupled CPET (diagonals in Figure 7) or through a stepwise transfer involving  and Q^•^^–^ intermediates. The coupled CPET mechanism offers the added advantage of bypassing these charged radical species and avoiding trapped intermediates in a reversed reaction (Figure 3C).

It can be hypothesized that Q redox chemistry catalyzed by other enzymes follows a similar mechanism. In particular, respiratory complexes I and II also contain Tyr and His side chains that coordinate the Q-head within their respective Q-binding sites.^52−54^ Based on the free energy profiles and analysis presented here, charged closed-shell intermediates such as Q^2–^, QH^–^, or QH^+^ are also unlikely to be formed within these other respiratory complexes.

Although not directly simulated in this study, it is plausible to speculate that the protons bound to H152 and PRA_*bL*_ are subsequently transferred to bulk water on the positive side of the membrane. H152 resides in the head of the Rieske domain, which is flexible and capable of moving to approach the cytochrome *c*_1_ heme center.^9^ This movement may expose H152 to bulk water, potentially facilitating proton release after oxidation of the FeS center.^43^ PRA_*bL*_ is located in a hydrated region directly in contact with bulk water.^20^ Consequently, the excess proton bound to this propionate can be readily released^48,55^ after heme *b*_*L*_ is oxidized by heme *b*_*H*_ (Figure 1A).^9^ These processes would reset the redox and protonation states of the metal cofactors and protonable groups in the Q_o_ site, enabling another catalytic cycle following the exchange of the produced quinone (Q^0^) for a new quinol substrate.

## Conclusions

The simulations presented here integrate hybrid QC/MM potentials, a global nonlinear reaction coordinate and free energy profiles to probe the energetics of possible PT reactions after quinol oxidation in the Q_o_ site of cytochrome *bc*_1_. Beyond classical MD simulations that revealed local hydration and possible proton wires in the Q_o_ site,^20^ the current qualitative analysis may energetically validate PT pathways and exclude trapped intermediates or highly dissipative reactions.

Key reactive groups and mechanisms of proton transfer were identified for Q redox reactions. The first PT is energetically feasible to Rieske H152 after one-electron oxidation of quinol, resulting in the formation of a monoprotonated semiquinol radical (QH^•^). The second PT from this radical intermediate to heme PRA_*bL*_ is thermodynamic and kinetic viable, supporting the role of heme *b*_*L*_ as a final PT acceptor.

Residue Y147 in cyt *b* is a necessary initial and transient proton acceptor, without which complete PT to intermediate (such as E295) and final (heme PRA_*bL*_) acceptors is hindered. Conversely, residues H276 and D278 were found unlikely to contribute as proton acceptors.

Simulations reveal that multiple and redundant proton wires facilitate the PT reactions, creating a proton conducting network within the Q_o_ site, that is also robust to single-point mutations. Combined with the previously identified flexibility of side chains and reactive binding modes of the Q-head,^20^ the network of PT pathways has a significant entropic contribution to the PT efficiency in the Q_o_ site.

The overall mechanism suggests that the quinol oxidation sequence proceeds via QH_2_ → QH^•^ → Q^0^, without charged closed-shell intermediates. This pathway avoids high-energy charged species and reinforces an energetically optimized process for concerted proton–electron transfer in the Q_o_ site. These findings may be applicable to other enzymes with similar active sites and Q redox chemistry, and may help to understand proton transfer reactions in quinol-quinone redox cycling.

## Data Availability

Initial configurations and sample pDynamo scripts with all simulation parameters were deposited online^35^ to enable full reproduction of this study. The pDynamo library version 3 used here is freely available on GitHub.^34^

## Supporting Information Available

Comparison of potential energies obtained with different QC methods; example of sampling and convergence of the computed free energy profiles; details of the three initial configurations used for QC/MM simulations; and supporting references (PDF)
